# Protective effect of Buddha’s Temple extract against tert-butyl hydroperoxide stimulation-induced oxidative stress in DF-1 cells

**DOI:** 10.5713/ab.23.0014

**Published:** 2023-02-27

**Authors:** Eun Hye Park, Sung-Jo Kim

**Affiliations:** 1 Division of Cosmetics and Biotechnology, College of Life and Health Sciences, Hoseo University, Baebang, Asan 31499, Korea

**Keywords:** Antioxidant, Buddha’s Temple, Cytoprotective, Free Fatty Acid, *Gallus gallus* DF-1 Fibroblast Cell, Reactive Oxygen Species, Tert-butyl Hydroperoxide (t-BHP)

## Abstract

**Objective:**

This study aimed to determine the protective efficacy of Buddha’s Temple (BT) extract against tert-butyl hydroperoxide (t-BHP)-induced oxidative stress in *Gallus gallus* chicken embryo fibroblast cell line (DF-1) and its effects on the cell lipid metabolism.

**Methods:**

In this experimental study, *Gallus gallus* DF-1 fibroblast cells were pretreated with BT 10^−7^ for 24 hours, followed by their six-hour exposure to t-BHP (100 μM). Water-soluble tetrazolium salt-8 (WST-8) assays were performed, and the growth curve was computed. The intracellular gene expression changes caused by BT extract were confirmed through quantitative polymerase chain reaction (qPCR). Flow cytometry, oil red O staining experiment, and thin-layer chromatography were performed for the detection of intracellular metabolic mechanism changes.

**Results:**

The WST-8 assay results showed that the BT pretreatment of *Gallus gallus* DF-1 fibroblast cell increased their cell survival rate by 1.08%±0.04%, decreased the reactive oxygen species (ROS) level by 0.93%±0.12% even after exposure to oxidants, and stabilized mitochondrial activity by 1.37%±0.36%. In addition, qPCR results confirmed that the gene expression levels of tumor necrosis factor *α* (*TNFα*), TIR domain-containing adapter inducing IFN-beta (*TICAM1*), and glucose-regulated protein 78 (*GRP78*) were regulated, which contributed to cell stabilization. Thin-layer chromatography and oil red O analyses showed a clear decrease in the contents of lipid metabolites such as triacylglycerol and free fatty acids.

**Conclusion:**

In this study, we confirmed that the examined BT extract exerted selective protective effects on *Gallus gallus* DF-1 fibroblast cells against cell damage caused by t-BHP, which is a strong oxidative inducer. Furthermore, we established that this extract significantly reduced the intracellular ROS accumulation due to oxidative stress, which contributes to an increase in poultry production and higher incomes.

## INTRODUCTION

Oxidative stress is a considerable contributor to the development of various human diseases. However, a number of antioxidant compounds can neutralize excessive free radicals, protect cells from their toxic effects, and prevent or treat diseases. Reactive oxygen and nitrogen species (ROS/RNS) are considered essential for various cell functions [1]. However, increased oxidant levels create an imbalance in the cell redox states that allows oxidative stress to overcome the capacity of the antioxidant defense networks leading to cell damage and even death. Previous reports have confirmed that oxidative stress is involved in the development of various diseases, including diabetes, neurodegeneration, cancer, and atherosclerosis. Although enzyme and non-enzyme defense systems detoxify the ROS/RNS already present in cells and suppress ROS/RNS generation, under certain pathological conditions [2] antioxidant defense systems are not able to offset the toxic effects of amplified oxidative stress [3,4]. This protective inability is due to the unavailability of a defense system against the harmful effects of oxidizing accelerators.

Therefore, targeting the imbalance of intracellular oxidant and antioxidant agents is an appropriate approach for the treatment of various secondary complications associated with oxidative stress-related diseases and disorders [5]. Enhancing the levels of exogenous antioxidants or activities can be effective as a therapeutic approach against the overproduction of ROS/RNS [6]. Reactive oxygen species are generated in the process of energy production necessary for maintaining life by using oxygen from the air [7]. Reactive oxygen species are known to directly or indirectly cause damage to the body by their involvement in a wide range of biological activities. Singlet oxygen (^1^O_2_) is produced by the reduction of triplet oxygen (^3^O_2_), which is the most stable form of this element [8,9]. If this oxidative stress persists, it damages DNA, causing cancer; unsaturated fatty acids are attacked, which are components of cell bio-membranes, and a peroxidation reaction is induced, leading to the accumulation of lipid peroxide in the body, resulting in skin aging, arteriosclerosis, stroke, diabetes, and tumor development [10–13]. Tert-butyl hydroperoxide (t-BHP) is an environmental toxicant widely used as an oxidative stress inducer that triggers ROS production under many conditions and stimulates cell death [14]. Due to the characteristics of t-BHP, it is widely used in simulation studies as an inducer of oxidative stress. After t-BHP penetration through the cell membrane, it is easily decomposed into alkoxyl and peroxyl radicals, generating ROS that cause damage to proteins, nucleic acids, and cell membranes, and promote lipid peroxidation, cytotoxicity, and apoptosis [15,16]. Antioxidant dietary supplements have received considerable attention due to their ability to combat the damage caused by oxidative stress. Research is ongoing on the potential application of antioxidants derived from natural sources as an adjuvant treatment of oxidative stress-caused disorders. In this regard, antioxidant effects and anticancer effects of natural bioactive compounds isolated from succulents have been reported [17,18].

In this study, we found that the *Crassula* ‘Buddha’s Temple (BT)’ extract from the succulent plant Crassaceae could maintain ROS, Mitochondria homeostasis and regulate lipid levels. The anti-inflammatory effects and mechanisms of action and the expression patterns of the bioactive substances in chicken embryo fibroblast cell line (DF-1) cells were previously investigated [19]. Our results can contribute to better understanding the response to BT treatment and the related gene expression in chicken fibroblast cells, and furthermore, through the resolution of lipid accumulation generated by stress in chicken fibroblast cells, improvement of muscle content, increase in body weight, and production of healthy chicken. Based on the effect, it presents the grounds that can contribute to the improvement of productivity of farm households.

## MATERIALS AND METHODS

### Preparation of Buddha’s Temple extract

Buddha’s Temple was mixed with 30 g of distilled water (DW) and extracted in an autoclave (Daihan Scientific, Wonju, Korea) at 110°C and 39.23 kPa for 15 minutes. Then, the aqueous phase was collected and filtered using a 0.22-μm cellulose-acetate filter (16534-K; Sartorius, Goettingen, Germany).

### Cell culture conditions

*Gallus gallus* DF-1 fibroblast cells were purchased from the American Type Culture Collection and supplemented with 10% (v/v) fetal bovine serum (TMS-013-BKR; Millipore, Burlington, MA, USA) and Dulbecco’s modified eagles medium supplemented with penicillin streptomycin (DMEM; 10–013-CVR; Corning, Corning, NY, USA) in a CO_2_ incubator (95% air and 5% CO_2_, 36.5°C).

### Treatment conditions and cell viability analysis

After culturing *Gallus gallus* DF-1 fibroblast cells, they were sub-cultured with 0.25% trypsin-ethylenediaminetetraacetic acid, inoculated into a 96-well plate (1×10^3^ cells/cm^2^), and cultured for 24 hours. The cells were pretreated with BT 10^−7^ dissolved in dH_2_O and incubated with t-BHP at a concentration of 10 μM for 6 hours. This experiment consisted of vehicle, BT 10^−7^, t-BHP 10 μM, and BT 10^−7^ + t-BHP 10 μM treatments.

To measure cell viability, water-soluble tetrazolium salt (WST)-8 cell viability assay and growth curve analysis were performed as previously described [4]. Briefly, (1×10^3^) DF-1 cells were seeded in a 96-well cell culture plate (SPL Life Sciences, Pocheon, Korea). To determine cell viability, WST-8 reagent (EZ Cytox Cell Viability Assay Kit; DoGenBio, Seoul, Korea) was added to each well, and the absorbance was measured at 450 nm using a microplate reader (Multi mode microplate reader, FilterMax F3).

### Quantitative real-time polymerase chain reaction

Total RNA extraction was performed using TRIzol reagent (15596018; Invitrogen, Carlsbad, CA, USA) following to the manufacturer’s instructions. cDNA was generated using the WizScript cDNA Synthesis Kit (W2202; Wizbiosolutions, Seongnam, Korea) with oligo-dT primer. Quantitative polymerase chain reaction (qPCR) next was performed using a Step OnePlus RT-PCR system (Applied Biosystems, Foster City, CA, USA) with specific primers (Table 1) and SYBR Green qPCR Mix (DQ485; BioFACT, Daejeon, Korea). The cycle threshold (Ct) value was confirmed using Step One software version 2.3 (Applied Biosystems, USA), and the mRNA fold-change value was determined using the 2 (−ΔΔCt) method [20].

### Thin-layer chromatography

Total lipid extraction was performed using the Bligh and Dyer method [21]. Briefly, an average of 1×10^6^ cells were lysed in water/chloroform/methanol = 1:2:0.8 (v/v/v) for 12 h at 4°C and then in water/chloroform/methanol = 2:2. The 1.8 (v/v/v) chloroform layer was concentrated for 1 hour at 4°C using a Speed Vac (EYELA, Kouto-Ku, Tokyo, Japan) and plated on a silica gel preparative thin-layer chromatography (TLC) plate (P46021; Analtech, Newark, DE, USA). TLC plates were developed in a solvent system of cyclohexane/ethyl acetate acid = 1:2 (v/v), and the lipids were visualized by incubation with 10% (v/v) sulfuric acid at 120°C. Real-time densitometry was performed, and the Rf value was established to determine the lipid type.

### Lipid staining and concentration measurement

Oil red O was used for lipid staining with minor modifications as previously described [6]. Briefly, 1×10^6^ *Gallus gallus* DF-1 fibroblast cells were seeded in a 60 mm cell culture dish. After 12 hours, the cells were pretreated with BT 10^−7^ for 24 hours and incubated for 6 hours with t-BHP (100 μM). Then, the cells were washed with phosphate buffered saline, fixed in 3.8% formaldehyde for 15 minutes, washed with 60% (v/v) isopropanol, air-dried, and oil red O stained (Sigma Aldrich, St Louis, MO, USA; 0.2-μm filtration, 0.3% [w/v] after washing 60% [v/v]) isopropanol-stained cells with DW, the oil red O staining was examined using a DMi8 fluorescence microscope (Leica, Deerfield, IL, USA), images were taken with LAS X (Leica, USA) and processed with Photoshop and the CC 2018 program.

### Data analysis and statistics

All results were obtained from three independent replicates (biological conditions, n = 3) and expressed as mean±standard deviation. Analysis was performed using GraphPad PRISM software version 9.4.1 (GraphPad Software, San Diego, CA, USA) and Microsoft Excel for Microsoft 365 MS (build 16.0. 14527.20270) (Microsoft, Redmond, WA, USA). The p-value was calculated using the method indicated in the figure legend p<0.05 was considered to indicate a statistically significant difference.

## RESULTS

### Crassula ‘Buddha’s Temple’ hot water extraction

**Figure 1:**
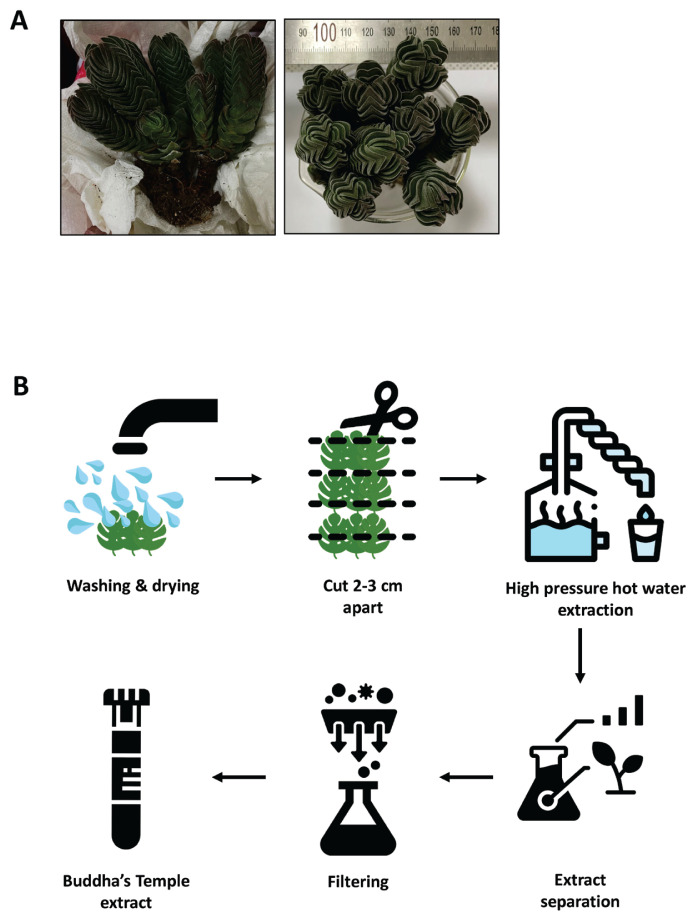
(A) The appearance of Buddha’s Temple. (B) The manufacturing process of the Buddha’s Temple extract. Buddha’s Temple stems and roots were washed with DW and cut into 2 to 3-cm long pieces. Then, 138 g of Buddha’s Temple stem and 414 mL of DW were added, and high-pressure hot water extraction was conducted for 15 minutes at 39.23 kPa and 110°C. Finally, to produce Buddha’s Temple extract, Buddha’s Temple stem and root were next removed and sterilized using a filter with a 0.22-μm pore size.

Buddha’s Temple was washed with DW, cut into 2 to 3 cm pieces, mixed with dH_2_O at a BT: dH_2_O ratio of 1:3, and extracted with high-temperature and high-pressure hot water for 15 minutes at 110°C and 39.23 kPa. The BT residue was removed, and a BT extract was prepared by sterilization filtration through a 0.22-μm pore size filter (Figure 1).

### Verification of DF-1 cytoprotective efficacy of Buddha’s Temple extract

**Figure 2:**
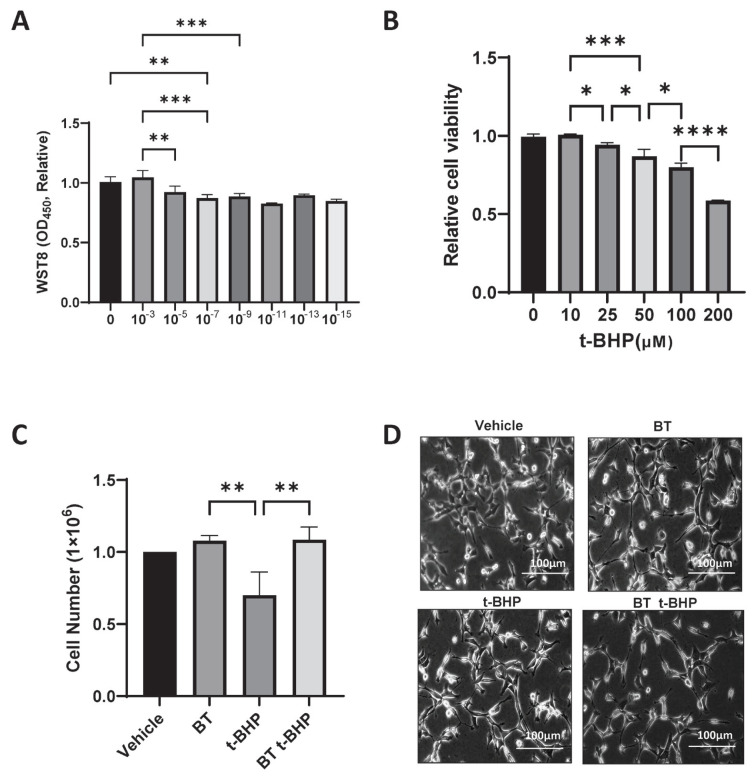
Effect of BT on the DF-1 cell damage induced by t-BHP. (A) Determination of the effects of various concentrations of Buddha’s Temple on DF-1 cells. The cell count (%) data are expressed as mean±SD. * p<0.05, ** p<0.01, *** p<0.001, *** p<0.0001 by two-way ANOVA with Tukey’s multiple comparison test. (B) Cell viability in DF-1 cells exposed to t-BHP for 6 hours (* p<0.05, ** p<0.01, ** p<0.001, ** p<0.001 by two-way ANOVA with Tukey’s multiple comparison test). (C) BT 10^−7^ cell viability analysis after 24-hour incubation in an incubator followed by 6-hour exposure to 10 Mm of t-BHP (* p<0.05 by two-way ANOVA with Tukey’s multiple comparison test.). (D) Microscopic observational morphological images of DF-1 cells exposed to t-BHP for 6 hours. The data are expressed as mean±SD. BT, Buddha’s Temple; DF-1, chicken embryo fibroblast cell line; t-BHP, tert-butyl hydroperoxide; ANOVA, analysis of variance; SD, standard deviation.

We investigated the effects of the concentration of the BT extract on the cell viability. A WST-8 assay was performed to determine the range to be used in the experiment. The determination of the cytoprotective efficacy of the BT extract on *Gallus gallus* DF-1 fibroblast cells confirmed that an optimal cell viability was achieved at a concentration of 10^−7^. Therefore, in this study, 10^−7^ was selected as the optimal concentration for measuring the cytoprotective effect of the BT extract (Figure 2A), and this concentration was applied in all subsequent experiments.

### Measurement of the viability of DF-1 cells treated with different t-BHP concentrations

The dose-response relationship between the viability of the DF-1 cells and the potent pro-oxidant t-BHP was evaluated. The effect of each concentration (10, 25, 50, and 100 μM) of t-BHP on *Gallus gallus* DF-1 fibroblast cells for 24 hours was assessed by WST-8 assays. We observed a t-BHP treatment-induced reduction in the viability of the *Gallus gallus* DF-1 fibroblast cells in a concentration-dependent manner; 100 μM of t-BHP, which induced optimal cell death, was used in our subsequent experiments (Figure 2B).

### Verification of the protective effect of the Buddha’s Temple extract against t-BHP-induced cytotoxicity

To investigate the cytoprotective effect of BT extract against t-BHP-induced damage to *Gallus gallus* DF-1 fibroblast cells, the previously identified BT was added to the treated cells at a concentration of 10^−7^, and after incubation for an additional 24 hours, treatment with 100 μM of t-BHP was conducted for 6 hours. Then, cell viability was measured by WST-8 assay. Under the experimental conditions, BT increased cell viability, and the cell viability damage by t-BHP was significantly reduced by 0.70%±0.16% compared to 1.08%±0.04% by BT treatment (Figure 2C). The cell viability in the BT treatment (10^−7^) in *Gallus gallus* DF-1 fibroblast cells was confirmed using an optical microscope, and no morphological differences were observed (Figure 2D).

### Buddha’s Temple reduces t-BHP-induced ROS production in DF-1 cells

**Figure 3:**
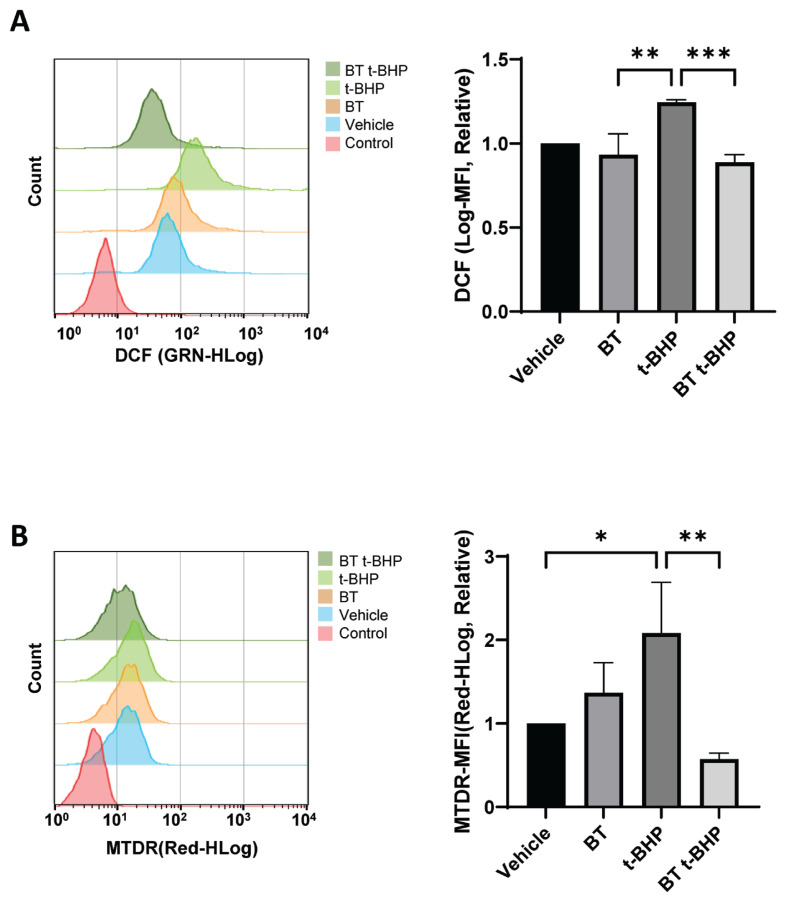
BT reduces the intracellular t-BHP-induced ROS levels in DF-1 cells. (A) After culturing in BT10^−7^ for 24 hours, the DF-1 cells were exposed to t-BHP for 6 hours, and the changes in the ROS levels were determined using flow cytometry with H2 DCFDA; * p<0.05 by two-way ANOVA with Tukey’s multiple comparison test. (B) After culturing in BT10^−7^ for 24 hours, the changes in the intracellular mitochondrial levels in DF-1 cells exposed to t-BHP for 6 hours were determined using MTDR dye. The values in the graph are expressed as mean±SD. BT, Buddha’s Temple; t-BHP, tert-butyl hydroperoxide; ROS, reactive oxygen species; DF-1, chicken embryo fibroblast cell line; H2 DCFDA, 2’,7’-dichlorodihydrofluorescein diacetate; ANOVA, analysis of variance; SD, standard deviation. The 24-hour BT treatment was followed by 6-hour t-BHP exposure; * p<0.05, ** p<0.01, *** p<0.001, **** p<0.0001 by two-way ANOVA with Tukey’s multiple comparison test.

To measure the changes in the intracellular ROS production, we examined the effect of BT on the ROS production in *Gallus gallus* DF-1 fibroblast cells treated with BT and t-BHP by flow cytometry using 2’,7’-dichlorodihydrofluorescein diacetate (DCF-DA). Our results confirmed that a concentration of 100 μM of t-BHP promoted ROS production in DF-1 cells, whereas BT extract inhibited the production of ROS (Figure 3A).

### Buddha’s Temple attenuates t-BHP-induced excessive mitochondrial stress in DF-1 cells

Mitochondria are essential for maintaining cell life activities and play an important role in regulating apoptosis that occurs during membrane permeability assessment [22]. They are critically important for cell survival, energy production, and ROS generation, which makes mitochondria one of the main targets for cancer treatment [23]. It is known that oxidative stress induces excessive activation of mitochondria, resulting in mitochondrial stress, leading to apoptosis [24]. To establish whether BT treatment affects homeostasis mechanisms such as mitochondrial activity stabilization, we measured the mitochondrial activity and confirmed the related gene expression changes. The mitochondrial activity was measured by flow cytometry analysis using MitoTracker Deep Red (MTDR; Thermo Fisher Scientific, Waltham, MA, USA). Although excessive mitochondrial activity was induced by t-BHP treatment, our results confirmed that BT attenuated the excessive mitochondrial stress induced by t-BHP in DF-1 cells (Figure 3B).

### Buddha’s Temple stabilizes the t-BHP treatment-induced mRNA expression level change

Oxidative stress can affect the expression levels of antioxidant genes. A number of diseases occur when ROS production exceeds a threshold up to which control is exerted by the antioxidant defense system. To further investigate the molecular mechanism of the neuroprotective effect of BT against oxidative damage induced by t-BHP, we investigated the mRNA expression of BT and t-BHP in DF-1 cells by qPCR.

**Figure 4:**
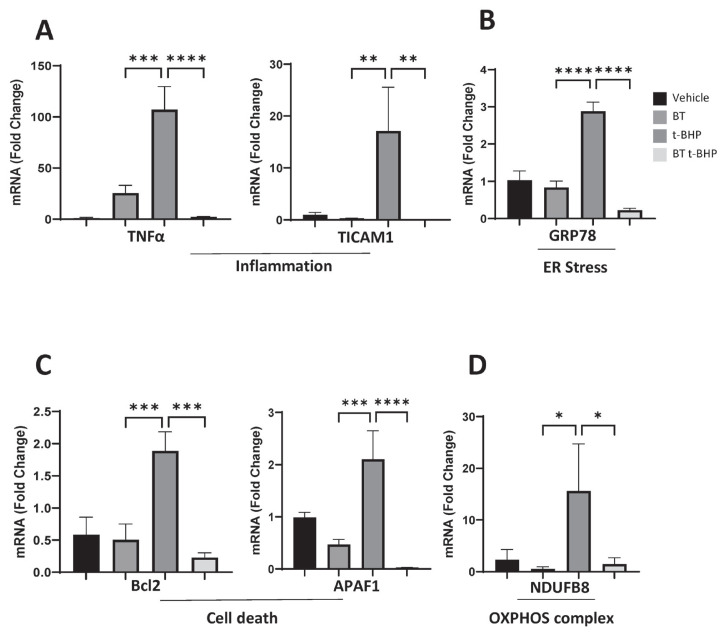
BT pretreatment regulates mRNA expression changes after t-BHP treatment. (A)–(E) DF-1 cells treated with BT for 24 hours and then exposed to t-BHP for 6 hours; the mRNA expression of the genes related to glycolysis was confirmed. BT, Buddha’s Temple; t-BHP, tert-butyl hydroperoxide; DF-1, chicken embryo fibroblast cell line. * p<0.05, ** p<0.01 *** p<0.001, **** p<0.0001 by two-way analysis of variance; with Tukey’s multiple comparison test.

Among the intracellular phenomena associated with oxidative damage, the expression of inflammatory genes of commonly known inflammation-related genes (tumor necrosis factor-alpha [*TNFα*] and TIR domain-containing adapter inducing IFN-beta [*TICAM1*]) was analyzed (Figure 4A), and the level of glucose-regulated protein 78 (GRP78), an endoplasmic reticulum (ER) stress factor, was evaluated. The expression of was confirmed (Figure 4B). In addition, the expression levels of cell death-related genes (B-cell lymphoma 2 [*Bcl-2*], apoptotic protease-activating factor 1 [*APAF1*]) (Figure 4C) and oxidative phosphorylation-related OXPHOS complex (NADH dehydrogenase (ubiquinone) 1 beta subcomplex assembly factor 8 [*NDUFB8*]) genes were determined (Figure 4D). Our results confirmed that BT effectively reduced the t-BHP-increased mRNA expression level of all gene expression patterns in DF-1 cells. Therefore, we confirmed that a cell protective effect was exerted by reducing cell damage.

### Buddha’s Temple downregulates the neutral lipid content in DF-1 cells

Fat accumulation is a key feature of diabetic kidney disease pathogenesis that is attributed to excessive lipid accumulation (hyperlipidemia) [25]. Renal lipotoxicity is characterized by the accumulation of excessive amounts of intracellular free fatty acids (FFAs) leading to the accumulation of potentially toxic metabolites such as diacylglycerol and ceramide [26]. Kidney damage induced by lipid accumulation occurs through several mechanisms, including the production of ROS and the release of proinflammatory and profibrotic factors [27]. In addition, the elevated levels of ROS affect the homeostasis of the intracellular lipid metabolism, interfering with lipid synthesis and degradation processes [28]. An increase in the ROS level decreases the mitochondrial activity and fatty acid oxidative degradation level, while neutral lipid aggregation and triglyceride production is promoted [29].

**Figure 5:**
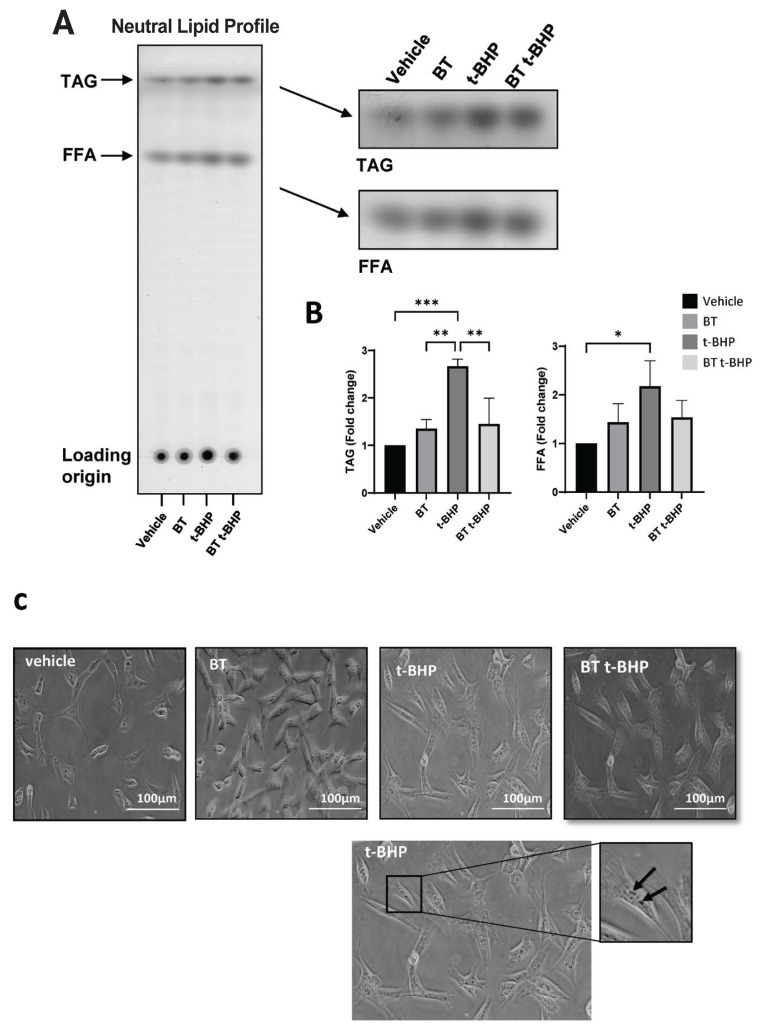
BT downregulates the neutral lipid content in DF-1 cells. (A) TLC of neutral lipids in DF-1 cells incubated with BT 10^−7^ for 24 hours. (B) Densitometry of TAG and FFA. Data are expressed as mean±SD. (C) The lipid accumulation of BT-treated cells was observed microscopically after 6-h exposure to t-BHP. BT, Buddha’s Temple; DF-1, chicken embryo fibroblast cell line; TLC, thin-layer chromatography; TAG, triacylglycerol; FFA, free fatty acid; SD, standard deviation; ANOVA, analysis of variance. * p<0.05, ** p<0.01 *** p<0.001, **** p<0.0001 by two-way ANOVA with Tukey’s multiple comparison test.

To assess the intracellular lipid droplet production, oil red O staining, which is known to react with neutral fats, was performed, and the effect of BT on neutral lipid production in DF-1 cells was established by qPCR and TLC (Figure 5C). High lipid levels lead to excessively elevated ROS amounts, resulting in cellular damage.

The TLC was performed to confirm the change in the lipid content caused by the pre-BT t-BHP treatment. The t-BHP treatment of DF-1 cells increased triacylglycerol (TAG) by 2.67%±0.15% and FFA by 2.17%±0.53% compared to the respective levels in the vehicle treatment. After pretreatment with BT, when t-BHP was treated, TAG was 1.45%±0.55% and FFA 1.54%±0.35% compared to vehicle, and the expression of excessively produced neutral lipids by t-BHP was suppressed (Figure 5A,5B).

## DISCUSSION

In this study, the ability of BT to protect cells from t-BHP-induced oxidative damage was investigated in DF-1 cells. We found that BT strongly reduced t-BHP-induced cytotoxicity and apoptosis. In addition, BT efficiently prevented t-BHP-induced mitochondrial dysfunction and oxidation of biomolecules in DF-1 cells. More specifically, it considerably suppressed the t-BHP-induced ROS overproduction in DF-1 cells. These results suggest that BT can protect DF-1 cells from t-BHP-induced cell damage by enhancing the activities of the endogenous enzyme antioxidant defense system.

In addition, mitochondria, the major intracellular ROS producers, are paradoxically sensitive to oxidative stress. Redox damage can easily lead to mitochondrial dysfunction by inhibiting mitochondrial enzymes involved in ATP production [30]. The disruption of oxidative phosphorylation directly affects the respiratory chain electron flux and reduces mitochondrial membrane permeability. Accompanied by increased electron leak in the mitochondrial respiratory chain, enhancing ROS production can put cells at risk of damage [31]. Destructive feedback loops in cells can be created, which can lead to the activation of intrinsic apoptotic pathways. Mitochondrial dysfunction, ROS overproduction, and increased cell death rates are important factors associated with pathological processes in humans and economically important domestic animals. In this study, we evidenced that the negative effects caused by lipid accumulation in fibroblast cells, as well as ROS and mitochondrial dysfunction generated after inducing strong oxidative stress in DF-1 cells, were reduced by BT pre-treatment.

Specifically, the t-BHP-treated DF-1 cells had increased expression of apoptosis genes (Bcl-2 4.16%±3.06%) and upregulated oxidative stress-related enzymes inflammation genes (*TNFα*, 120.02%±0.15%; *TICAM1*, 22.64%±17.70%), ER stress gene (*GRP78*, 2.89%±0.64%), cell death gene (*Bcl2*, 4.16%±3.06%; *APAF1*, 2.17%±0.77%), and OXPHOS complex gene (*NDUFB8*, 13.83%±16.89%). The expression levels of these genes in the BT + t-BHP treatment were considerably lower than those in the vehicle treatment (*TNFα*, 2.39± 0.99; *TICAM1*, 0.03%±0.01%; *GRP78*, 0.23%±0.09%; *Bcl2*, 0.43%±0.13%; *APAF1*, 0.03%±0.00%; *NDUFB8*, 0.65%± 0.08%). The results showed that the mitochondrial activity and fatty acid oxidative degradation levels were reduced along with the decrease in the ROS levels increased by the BT treatment. These results confirm that BT downregulates lipid accumulation by regulating the antioxidant, anti-apoptosis, and anti-inflammation capacity of DF-1 cells, which is a mechanism for the alleviation of t-BHP-induced oxidative stress damage.

## Figures and Tables

**Table 1:** Lists of primers used to perform quantitative polymerase chain reaction

Target gene (accession number of NCBI)	Primer type 5′ to 3′	Sequence
*TNFa* (MF000729.1)	Forward	GGTTCGAGTCGCTGTATCAGG
	Reverse	ACTCCCACCACCCCAAAATA
*TICAM1* (NM_001081506.1)	Forward	CACATCTGCTCAGGTGGGTC
	Reverse	GGATGATGATGGAACGGGCA
*Bcl-2* (NM_205339.2)	Forward	GGATGGGATGCCTTTGTGGA
	Reverse	AGAGTGATGCAAGCTCCCAC
*APAF1* (XM_416167.6)	Forward	GGACGACAGCCTTTTCCTGA
	Reverse	TCTTTGAGCCCGTAGCTTGG
*GRP78* (NM_205491.1)	Forward	TCGGCTAACACCAGAGGAGA
	Reverse	CGGGCATCAATGCGTTCTTT
*NDUFB8* (NM_001006502.1)	Forward	GTGGGACCGAAGCAGTATCC
	Reverse	CGGTGGCTCTTTATTGGGGT

*TNF a*, tumor necrosis factor-alpha; TICAM1, TIR domain-containing adapter inducing IFN-beta*; Bcl-2*, B-cell lymphoma 2; *APAF1*, apoptotic protease-activating factor 1; *GRP78*, glucose-regulated protein 78; *NDUFB8*, NADH dehydrogenase (ubiquinone) 1 beta subcomplex assembly factor 8.
